# Lamotrigine ODT-Induced Seizure in a 3-Year-Old Child after Accidental Ingestion

**DOI:** 10.1155/2019/2675931

**Published:** 2019-01-13

**Authors:** Ashley Griswold, Briana Tully, Kenneth Katz, Gillian Beauchamp, Matthew Cook, Robert Cannon

**Affiliations:** ^1^Department of Pediatrics, Lehigh Valley Health Network/University of South Florida Morsani College of Medicine, Cedar Crest Boulevard & I-78, Allentown, PA 18103, USA; ^2^Department of Emergency and Hospital Medicine, Lehigh Valley Health Network/University of South Florida Morsani College of Medicine, Cedar Crest Boulevard & I-78, Allentown, PA 18103, USA

## Abstract

Lamotrigine is a new generation antiepileptic which blocks sodium channels and can cause significant toxicity in overdose. A case of a three-year-old child who suffered a seizure and required endotracheal intubation after accidental lamotrigine ingestion is presented. The lamotrigine concentration measured 23.2 mcg/mL which is the highest reported after accidental pediatric exposure. A review of the literature regarding pediatric lamotrigine poisoning is also included.

## 1. Introduction

Lamotrigine was developed in 1994 and is prescribed for both partial and generalized seizures as well as bipolar disorder. The drug inhibits seizure activity primarily through voltage-gated sodium channel blockade. It also acts presynaptically as an inhibitor of glutamate and aspartate. In the postsynaptic setting, it diminishes excitability by inhibiting sodium conductance and may also reduce high-voltage activated calcium currents [1–3]. Unlike other anticonvulsants, lamotrigine has little or no effect on serotonergic, dopaminergic, or adrenergic receptors [2, 4]. Reported adverse effects with therapeutic use include nausea, vomiting, diplopia, dizziness, ataxia, tremor, and urinary tract infections. Most effects are mild to moderate in intensity and do not lead to discontinuation [1, 2]. More serious adverse events are dermatologic, such as Stevens-Johnson Syndrome, which appears to be more common in children than adults [2].

## 2. Case Presentation

A three-year-old, 14.5 kg boy, without medical history and prescribed no medications, presented to the emergency department (ED) within an hour of ingesting between seven and sixteen oral disintegrating 25 mg lamotrigine tablets and one to six 0.5 mg clonazepam tablets from his brother's pill organizer.

Emergency Medical Services arrived at the home within 20 minutes of ingestion and found the patient minimally responsive and arousable only to painful stimuli with poor respiratory effort. Bag-valve-mask ventilation was provided. An intraosseous (IO) line was established, and the patient was transported to the ED.

In the ED, the child had a Glasgow Coma Scale of 3 and minimal independent respiratory effort. Vital signs included heart rate of 100 beats per minute, pulse oximetry 100% (100% oxygen via bag-valve mask), temperature 35.7°C (96.2°F), and blood pressure 92/43 mmHg. The child then developed a tonic-clonic seizure which was treated with 1 mg IO lorazepam. The patient was endotracheally intubated for airway protection and admitted to the pediatric intensive care unit (PICU) for further monitoring and care. An electrocardiogram (QRS interval of 86ms), complete blood count, serum chemistries, serum acetaminophen, salicylate and ethanol concentrations were all unremarkable.

Serum lamotrigine concentrations measured 23.2 mcg/mL and 18.5 mcg/mL approximately three and 24 hours after ingestion, respectively. Serum liquid chromatography-mass spectrometry detected the following: acetaminophen, lamotrigine, 7-aminoclonazepam, midazolam, alpha-hydroxymidazolam, and lorazepam. This result is consistent with both ingestion of lamotrigine and clonazepam, as well as iatrogenic administration of lorazepam, midazolam, and acetaminophen.

While in the PICU, the child demonstrated mild hyperkinesia and periods of agitation. He was extubated on hospital day (HD) one, and these symptoms resolved over the subsequent 36 hours. Ultimately, the patient was discharged in normal condition on HD five.

## 3. Discussion

Lamotrigine poisoning in children is very rare. Seven identified cases of patients describe seizures, as well as hyperkinesia and ataxia following ingestion [3, 5–10]. These case reports of acute lamotrigine poisoning in pediatric patients were identified through literature review (Table 1). Case reports of lamotrigine intoxication in children were identified by searching PubMed using the following search terms: lamotrigine, lamictal, overdose, poisoning, pediatric, seizure, and adverse reactions. Case reports describing adult patients with adverse reactions as well as adverse reactions resulting from therapeutic dosing or chronic ingestion were excluded. Three cases of pediatric lamotrigine poisoning which included serum concentrations were identified. The highest documented serum concentration was measured in a 12-day-old boy who was being repeatedly poisoned while in the hospital and not after a single, accidental ingestion as in the case described in this report [5].

Lamotrigine is rapidly absorbed in the stomach with a bioavailability of approximately 98%. The drug is 50% plasma protein-bound, undergoes hepatic metabolism via glucuronidation, and is mainly eliminated by the kidneys with an elimination half-life of approximately 29 hours [1, 11]. Oral disintegrating tablets and regular immediate release oral tablets are bioequivalent [11]. Typically, the maximum lamotrigine dosage for maintenance in the pediatric age group is a maximum of one to five mg/kg/day divided into one or two doses. The largest potential dose consumed in our case was five times the maximum maintenance dosage for his age group [7].

Lamotrigine poisoning can cause seizures, behavioral disturbances, and cardiotoxicity. Seizures generally occur between 20 minutes and three hours after ingestion and have been described as myoclonic, tonic- clonic, and status epilepticus (Table 1). Hyperkinetic arm motion, dysconjugate gaze, and ataxic gait were evident on physical examination in this case. Severe ataxia and dysmetria have been reported and attributed to inhibition of GABA release [12]. Oculogyric crisis may occur from alteration of dopaminergic tone [12]. Neurologic effects generally resolve over 12 to 48 hours. While there have been no reported cases of cardiotoxicity in the pediatric population, this is a known risk of lamotrigine intoxication causing ventricular dysrhythmias in adults.

Treatment of lamotrigine poisoning comprises airway support and the use of titrated benzodiazepines, barbiturates, or propofol to ameliorate seizure and motor activity [13]. Administration of intravenous sodium bicarbonate is indicated for prolonged QRS interval or ventricular dysrhythmias. Intralipid emulsion administration with clinical improvement was reported in a case of a patient suffering from combined lamotrigine and bupropion toxicity [14]. Measurement of serum lamotrigine concentrations may be used to confirm exposure but may not be readily available; therefore, empiric treatment as outlined above is recommended.

Pediatric lamotrigine poisoning can cause severe toxicity, including abnormal motor activity, seizures, and possible cardiac dysrhythmias. The mainstay of treatment is empiric and supportive with attention to the airway and control of seizures.

## Figures and Tables

**Table 1:** Case reports of pediatric seizures following lamotrigine ingestion.

Reference	Age, sex	Dose, formula	Presentation	Drug concentration	Management	Outcome
Present case	3-year-old male	Up to 27.6 mg/kg in ODT tablets	Acute: 1 seizure <1 minute in duration within 1 hour with myoclonic activity, dysconjugate gaze, coma	23.2 mcg/mL (3-4-hour level) & 18.5 mcg/mL (24-hour level)	Lorazepam, intubation	Discharged well after 72 hours

Grosso (2016)	3.2-year-old male	1600 mg total (wt not given) 200 mg tablets	Acute: tonic-clonic status epilepticus upon presentation. After 30 min postictal pattern	28.4 mg/L after 10 hours of fluids	Rectal diazepam x 2, IVP midazolam, then cont infusion	Discharged at approx 72 hrs. Hyperphagia still present at discharge

Moore (2013)	1-year-old female	Unknown quantity	Acute: initially agitated and crying with intermittent myoclonus of her extremities	18 mg/L at 8 h and 11.8 mg/L at 19 h	Lorazepam	Discharged well after 19 hours

Close (2010)	2-year-old male	Up to 43 mg/kg tablet	Acute: 2 seizures of 5-second duration within 3 hours with ataxia, drowsiness, nystagmus, hyperreflexia vomiting	Not available	Diazepam	Discharged well after 12 hours

Thundiyil (2007)	19-month-old male	Unknown amount of a chewable tablet	Acute: 2 seizures of <10-second duration in 1 hour with irritability, vomiting, sinus tachycardia	20.3 mg/L (1-hour level)	Activated charcoal	Discharged well after 24 hours

Willis (2007)	12-day-old male	Not available	Acute: repeated poisoning (including in the hospital); 2 seizures (9 days apart), with arm movements, irritability, poor interaction, poor feeding	35 mg/L (2 days after the second seizure)	Lorazepam, phenytoin, paraldehyde	19-day admission with seizures at days 4 and 13, discharged well to child protection

Lofton (2004)	3-year-old female	Not available	Acute: multiple seizures with vomiting	Not available	Not available	Not available

Briassoulis (1998)	2-year-old male	62 mg/kg tablet	Acute: 2 seizures (15- and 5-minute duration) within 90 minutes with muscle weakness and incoordination, ataxia, tremor, hypertonia	3.8 mg/L (2-hour level)	Midazolam, gastric lavage, activated charcoal	Discharged well after 48 hours

Modified from the following: Close BR, Banks CJ: Seizures secondary to lamotrigine toxicity in a two-year-old. Ann Pharmacother 2010;44:1112-5.
